# Frailty Matters: Validation of an Automated Electronic Short Physical Performance Battery (eSPPB) for Predicting 30-Day Mortality in Hospitalized Cardiovascular Patients—A Step-by-Step Study

**DOI:** 10.3390/jcm15083093

**Published:** 2026-04-17

**Authors:** Lidia López García, Dohong Kim, Seongjun Yoon, Juan Carlos Gómez Polo, José Antonio Espín Faba, Isidre Vila Costa, Julián Pérez Villacastín Domínguez

**Affiliations:** 1 Cardiovascular Institute, Hospital Clínico San Carlos, 28040 Madrid, Spain; jc.gomezpolo@gmail.com (J.C.G.P.); josespin@ucm.es (J.A.E.F.); i.vilacosta@gmail.com (I.V.C.); julian.perez-villacastin@salud.madrid.org (J.P.V.D.); 2 Faculty of Nursing, Physiotherapy and Podiatry, Complutense University of Madrid, 28040 Madrid, Spain; 3 Dyphi Research Institute, Daejeon 34134, Republic of Korea; luis.kim@dyphi.com (D.K.); seongjun@dyphi.com (S.Y.)

**Keywords:** frailty, short physical performance battery, eSPPB, cardiovascular disease, digital health, mortality, geriatric cardiology

## Abstract

**Background:** Frailty is a major determinant of adverse outcomes in older adults with cardiovascular disease. Automated digital tools may facilitate routine frailty assessment in hospital settings; however, their validity and prognostic relevance in acutely hospitalized patients remain insufficiently established. **Methods:** In this prospective cohort study, 113 hospitalized cardiology patients underwent frailty assessment using both manual Short Physical Performance Battery (mSPPB) and an automated electronic SPPB (eSPPB) system. Agreement between methods was evaluated using Pearson correlation, intraclass correlation coefficients (ICCs), and Bland–Altman analysis. Frailty was defined as SPPB < 5. The association between frailty and 30-day mortality was assessed using logistic regression and Kaplan–Meier survival analysis. **Results:** Seventeen patients (15.0%) were classified as frail. Automated and manual SPPB scores were highly correlated (r = 0.994, *p* < 0.001) and demonstrated good agreement (ICC = 0.80). Bland–Altman analysis showed a mean difference of −1.63 points (95% limits of agreement −4.41 to 1.16). Frailty was associated with significantly higher 30-day mortality (17.6% vs. 2.1%, *p* = 0.009), corresponding to a tenfold increase in mortality odds (OR 10.07; 95% CI 1.5–67.5). An exploratory model showed apparent discriminative performance (AUC 0.83; 95% CI 0.71–0.95). **Conclusions:** Automated eSPPB demonstrated good agreement with manual assessment and was significantly associated with short-term mortality in hospitalized cardiovascular patients. These findings support the validity and potential clinical utility of automated frailty assessment for risk stratification in acute cardiology settings.

## 1. Introduction

Frailty is a multidimensional clinical syndrome characterized by diminished physiological reserve and increased vulnerability to stressors, resulting in a reduced ability to maintain homeostasis during acute illness or physiological challenges. It is increasingly recognized as a major determinant of adverse health outcomes in older adults, including prolonged hospitalization, functional decline, institutionalization, and mortality [1,2].

Frailty reflects cumulative declines across multiple organ systems, including musculoskeletal, cardiovascular, and neurological domains, and is closely linked to sarcopenia, chronic inflammation, and impaired energy metabolism. Cardiovascular disease and frailty frequently coexist and interact in a bidirectional manner. Cardiovascular conditions such as heart failure, coronary artery disease, and arrhythmias can accelerate the development of frailty through mechanisms including reduced cardiac output, impaired tissue perfusion, systemic inflammation, and decreased physical activity [3,4,5].

Conversely, frailty itself is independently associated with worse cardiovascular outcomes, including increased mortality, longer hospital stays, higher readmission rates, and poorer recovery following cardiovascular interventions. Frailty has therefore emerged as a critical factor in risk stratification and clinical decision-making in cardiovascular medicine, particularly in older and hospitalized patients. Accurate and timely identification of frailty is essential for optimizing clinical management. Among available assessment tools, the Short Physical Performance Battery (SPPB) is one of the most widely validated and clinically accepted instruments for evaluating physical frailty [6,7].

The SPPB assesses lower-extremity function through three standardized components: standing balance, gait speed over a short distance, and repeated chair stands. These objective measures provide a composite score reflecting physical performance and functional reserve. Numerous studies have demonstrated that lower SPPB scores are strongly associated with increased risk of disability, hospitalization, institutionalization, and mortality across diverse clinical populations [8].

Despite its strong prognostic value and widespread validation, routine implementation of the SPPB in hospital settings remains limited. Manual administration requires trained personnel, standardized timing procedures, and careful observation, which can introduce variability and increase staff workload [9].

In busy inpatient environments, competing clinical priorities and limited staffing may reduce the feasibility of systematic frailty assessment. Furthermore, manual measurement may be subject to interobserver variability and timing inaccuracies, potentially affecting reproducibility and scalability.

Recent advances in digital health technologies have created opportunities to automate physical performance assessment using sensor-based systems. Automated measurement platforms can capture motion parameters objectively, reduce observer dependency, and improve measurement consistency. These technologies may enable more efficient and scalable frailty screening, particularly in high-volume clinical settings such as cardiology wards.

AndanteFit is a novel digital platform designed to automate SPPB assessment using integrated LiDAR-based motion sensors and software-guided scoring algorithms. The system automatically measures gait speed, balance, and sit-to-stand performance, generating standardized electronic SPPB (eSPPB) scores without requiring manual timing or subjective interpretation. By reducing observer variability and simplifying workflow, automated assessment has the potential to facilitate routine frailty screening in clinical practice. Previous studies have demonstrated the feasibility of sensor-based SPPB measurement in community-dwelling populations, but evidence regarding its performance and prognostic value in hospitalized cardiovascular patients remains limited [10]. However, its validity and prognostic relevance in acutely hospitalized patients had not been fully evaluated.

Importantly, the clinical utility of automated frailty assessment depends not only on measurement accuracy but also on its ability to provide meaningful prognostic information. Frailty assessment is most valuable when it contributes to early risk stratification, informs clinical decision-making, and identifies patients at increased risk of adverse outcomes. However, the validity and prognostic relevance of automated eSPPB systems in acutely hospitalized cardiology patients have not been fully established.

Therefore, the aim of this study was to evaluate the validity and prognostic utility of an automated eSPPB system in hospitalized cardiovascular patients. Specifically, we sought to assess agreement between automated and manual SPPB measurements, describe clinical and functional factors associated with frailty, and examine the association between frailty status and short-term mortality. We hypothesized that automated eSPPB would demonstrate strong agreement with manual SPPB and provide clinically meaningful prognostic information in hospitalized cardiology patients.

## 2. Materials and Methods

### 2.1. Study Design and Setting

This was a prospective observational cohort study conducted at the Cardiovascular Institute of Hospital Clínico San Carlos, Madrid, Spain, between February and May 2025.

### 2.2. Participants

A total of 113 consecutive hospitalized cardiology patients were included. Eligible participants were clinically stable adults admitted with primary cardiovascular diagnoses. Exclusion criteria included hemodynamic instability, inability to stand or walk safely, or severe cognitive impairment preventing test completion.

### 2.3. Frailty Assessment

Frailty was assessed using:

#### 2.3.1. Manual SPPB (mSPPB)

Manual SPPB was performed by trained clinical staff according to standardized protocols. Gait speed was measured over a 4 m course, and participants were instructed to walk at their usual pace. Sit-to-stand time was measured using a standard chair without armrests, and participants were instructed to stand up and sit down five times as quickly as possible without using their arms.

#### 2.3.2. Automated SPPB (eSPPB)

Automated electronic SPPB (eSPPB) assessments were performed using the AndanteFit system Automated measurements were performed using the AndanteFit digital system, which uses integrated motion sensors and proprietary algorithms to automatically detect movement initiation, gait speed, and sit-to-stand transitions. The system provides automated scoring based on standard SPPB criteria, eliminating the need for manual timing or observer interpretation.

The AndanteFit device (Dyphi Research Institute, Seoul, Republic of Korea) uses LiDAR-based motion detection sensors. The device was positioned approximately 2 m in front of the participant and calibrated prior to each measurement according to manufacturer instructions.

Participants performed the gait speed test over a flat, unobstructed 4 m course. Two trials were conducted, and the best performance was used for analysis. A standard chair with seat height of approximately 45 cm was used for sit-to-stand testing.

The SPPB includes:

1. Standing balance
2. 4 m gait speed
3. Five-repetition sit-to-stand

Total score range: 0–12.

Frailty classification:

- Frail: SPPB < 5
- Pre-frail: 5–10
- Non-frail: >10

### 2.4. Outcome Measure

The primary outcome was all-cause mortality within 30 days of hospital admission.

### 2.5. Statistical Analysis

Continuous variables were expressed as mean ± standard deviation and categorical variables as frequencies and percentages. Agreement between manual and automated SPPB scores was assessed using Pearson correlation coefficients. Comparisons between frailty groups were performed using ANOVA for continuous variables and Chi-square tests for categorical variables. Logistic regression analysis was used to explore associations between clinical and functional variables and frailty status.

Model discrimination was assessed using the area under the receiver operating characteristic curve (AUC). The 95% confidence interval for the AUC was estimated using the Hanley and McNeil method. Kaplan–Meier survival analysis and log-rank testing were used to compare mortality between frailty groups. Agreement between manual and automated SPPB scores was further evaluated using the intraclass correlation coefficient (two-way random effects, absolute agreement) and Bland–Altman analysis.

Because frailty was defined using the SPPB score, the regression analyses including SPPB component measures (gait speed and sit-to-stand time) were considered exploratory and hypothesis-generating, and were intended to describe within-cohort associations rather than establish independent predictive effects.

A *p*-value < 0.05 was considered statistically significant.

### 2.6. Ethical Approval

The study was approved by the Institutional Ethics Committee of Hospital Clínico San Carlos (Approval No. 24/847-E; 20 February 2025). All participants provided written informed consent.

## 3. Results

### 3.1. Baseline Characteristics

A total of 113 hospitalized cardiology patients were included in the study. The mean age was 74.2 ± 11.7 years, and 57 participants (50.4%) were female. The average gait speed was 0.88 ± 0.24 m/s, and the mean time required to complete the five-repetition sit-to-stand test was 16.2 ± 5.5 s.

The distribution of primary cardiovascular diagnoses in the overall cohort included heart failure (n = 30), coronary artery disease (n = 20), angina (n = 9), myocardial infarction (n = 8), acute coronary syndromes (n = 5), pacemaker/ICD/ventricular tachycardia (n = 7), syncope (n = 4), and other unspecified cardiovascular conditions (n = 10). Among frail patients, heart failure was the most frequent diagnosis; however, no formal statistical comparison between diagnostic categories and frailty status was performed due to the limited sample size within subgroups.

### 3.2. Frailty Distribution and Functional Performance

Based on SPPB scores, 17 patients (15.0%) were classified as frail (SPPB < 5), 60 (53.1%) as pre-frail (SPPB 5–10), and 36 (31.9%) as non-frail (SPPB > 10) (Table 1).

Frailty status was significantly associated with age and functional performance measures. Frail patients were older (79.1 ± 10.5 years) compared with pre-frail (76.7 ± 11.2 years) and non-frail patients (68.4 ± 12.1 years; *p* < 0.01).

Marked differences were observed in physical performance. Frail patients demonstrated substantially slower gait speed (0.52 ± 0.10 m/s) compared with pre-frail (0.85 ± 0.18 m/s) and non-frail patients (1.03 ± 0.21 m/s; *p* < 0.001). Similarly, sit-to-stand time was significantly prolonged in frail individuals (22.8 ± 6.0 s) compared with pre-frail (16.7 ± 4.5 s) and non-frail patients (13.2 ± 3.2 s; *p* < 0.001).

Although frailty was more frequent among women (75.0%), sex differences did not reach statistical significance (*p* = 0.15).

### 3.3. Agreement Between Manual and Automated SPPB

Automated eSPPB scores demonstrated good agreement with manual SPPB measurements. Pearson correlation analysis revealed an extremely strong positive correlation between methods in the overall cohort (r = 0.994, *p* < 0.001). Similarly, strong agreement was observed within the frail subgroup (r = 0.961, *p* < 0.001).

These findings indicate that automated eSPPB provides highly consistent measurements compared with manual assessment, supporting its validity as a tool for objective functional evaluation in hospitalized cardiology patients.

Intraclass correlation analysis showed good agreement between methods, with an ICC of 0.80, indicating strong reliability of automated measurements.

**Figure 1:**
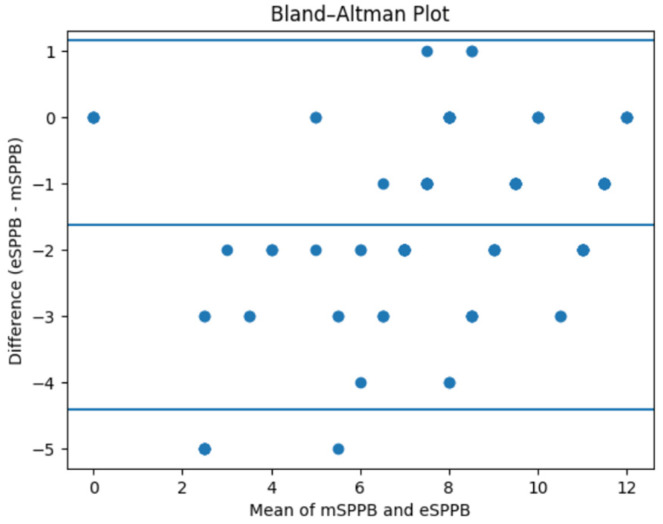
Bland–Altman plot showing agreement between manual and automated SPPB scores.

Bland–Altman analysis revealed a mean difference of −1.63 points (eSPPB − mSPPB), indicating a modest systematic tendency of the automated system to yield slightly lower scores than manual assessment. The 95% limits of agreement ranged from −4.41 to +1.16 points (Figure 1).

These findings suggest good overall agreement between methods, although a small systematic bias toward lower automated scores was observed.

### 3.4. Factors Associated with Frailty Status

Exploratory analyses were performed to examine associations between clinical and functional variables and frailty status within the cohort. Because frailty was defined using the SPPB score, which includes gait speed and sit-to-stand performance, these analyses were not intended to identify independent predictors but rather to describe within-cohort associations.

Slower gait speed was strongly associated with frailty status (β = −2.5, *p* < 0.001) (Table 2). Longer sit-to-stand time also showed an association with frailty risk, although this association did not reach conventional statistical significance (β = 0.14, *p* = 0.06). Age was not associated with frailty after adjustment for functional measures (β = 0.08, *p* = 0.21). Female sex was not significantly associated with frailty status (β = 0.45, *p* = 0.12).

**Figure 2:**
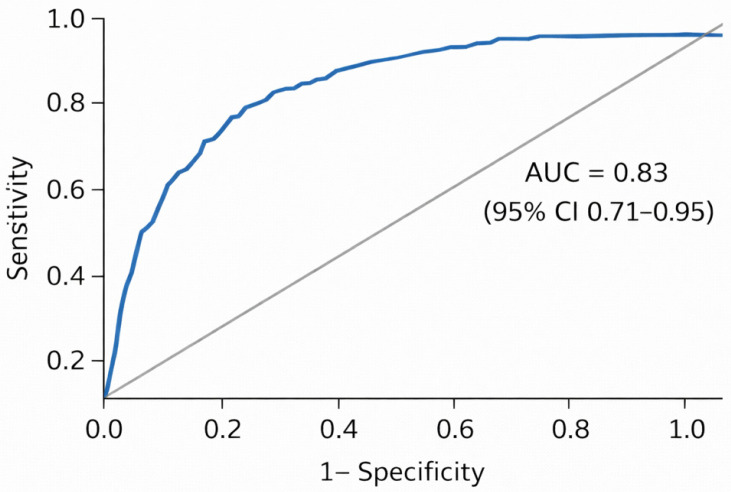
Receiver operating characteristic (ROC) curve for identification of frailty (AUC = 0.83; 95% CI: 0.71–0.95).

This exploratory model showed apparent discrimination within the study cohort (AUC 0.83; 95% CI 0.71–0.95); however, given the conceptual overlap between the included variables and the frailty definition, this result should be interpreted with caution and not as evidence of independent discriminative performance (Figure 2).

In exploratory analyses, the following variables were associated with frailty status within the cohort:

### 3.5. Thirty-Day Mortality

During the 30-day follow-up period, five deaths occurred (4.4%). Mortality was higher among frail patients than among non-frail patients. Specifically, three of 17 frail patients (17.6%) died within 30 days, compared with two of 96 non-frail patients (2.1%). Frailty was associated with increased odds of 30-day mortality (OR 10.07; 95% CI 1.54–65.69; Fisher’s exact *p* = 0.024) (Table 3). Kaplan–Meier survival analysis also demonstrated lower survival probability among frail patients (log-rank *p* = 0.009).

When frailty was defined using automated eSPPB scores, a similar association with 30-day mortality was observed. Specifically, three of 22 patients classified as frail according to eSPPB (13.6%) died within 30 days, compared with two of 91 non-frail patients (2.2%). Frailty defined by eSPPB was associated with increased odds of mortality (OR 6.96; 95% CI 1.08–44.7; Fisher’s exact *p* = 0.041).

**Figure 3:**
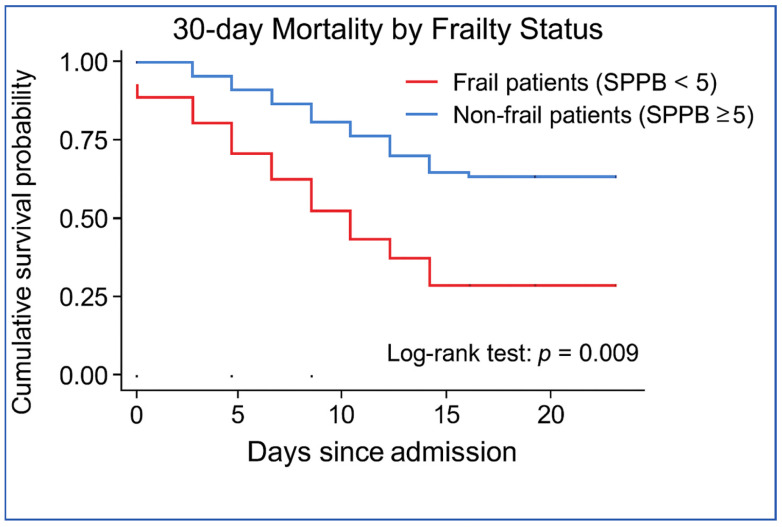
Kaplan–Meier survival curves according to frailty status.

Kaplan–Meier survival analysis demonstrated significantly lower survival probability among frail patients (log-rank *p* = 0.009) (Figure 3). The log-rank test confirmed a statistically significant difference in survival between frail and non-frail groups.

Frail patients experienced a noticeably lower survival rate over the 30-day period. The SPPB demonstrated significant association with short-term mortality.

## 4. Discussion

### 4.1. Principal Findings

In this prospective cohort study of hospitalized cardiology patients, we found that the automated eSPPB scores showed extremely strong correlation with manual measurements and demonstrated good agreement (ICC = 0.80), supporting the validity of this digital tool in hospitalized cardiovascular patients.

Importantly, frailty identified using SPPB was strongly associated with short-term mortality. Frail patients exhibited a tenfold increase in the odds of 30-day mortality compared with non-frail individuals. Although the number of events was limited, this finding underscores the clinical relevance of objective physical performance assessment for early risk stratification in hospitalized cardiovascular patients. These results are consistent with prior studies showing that lower SPPB scores are strongly associated with adverse outcomes, including mortality, rehospitalization, and functional decline in older adults with cardiovascular disease [11,12].

### 4.2. Clinical Relevance

Frailty is increasingly recognized as a key determinant of outcomes in patients with cardiovascular disease. Prior studies have demonstrated that reduced physical performance, particularly slower gait speed and impaired lower-extremity function, is associated with increased mortality, hospitalization, and functional decline. Our findings extend this evidence by demonstrating that automated frailty assessment using a digital system provides comparable measurements to traditional manual assessment and retains prognostic relevance.

Although Pearson correlation demonstrated an almost perfect linear association between manual and automated measurements, this metric reflects the strength of relationship rather than agreement. The ICC provides a more appropriate estimate of agreement and indicated good, but not perfect, reliability. The Bland–Altman analysis further identified a small systematic bias, with automated scores tending to be slightly lower. From a clinical perspective, this suggests that while eSPPB is suitable for ranking patients and identifying frailty, caution may be warranted when interpreting absolute values near diagnostic thresholds.

The strong correlation observed between manual and automated SPPB measurements suggests that digital tools can accurately capture clinically relevant functional parameters. Automation may reduce observer variability and improve standardization, potentially facilitating broader implementation of frailty screening in busy hospital settings where time and personnel constraints often limit routine functional assessment [12]. A recent review by Isaradech et al. highlighted the growing role of digital technologies in frailty detection, particularly those leveraging gait analysis, balance metrics, and upper-extremity motion [13]. However, many of these tools remain research prototypes or require wearable sensors, limiting their scalability and clinical integration. In contrast, eSPPB is fully automated, and based on a validated clinical scale (SPPB), making it more feasible for bedside use in acute care settings [14].

Recent validation studies of electronic SPPB systems, including kiosk-based and multi-sensor platforms, have primarily focused on community-dwelling older adults and feasibility outcomes rather than short-term clinical prognosis. In contrast, our study extends this evidence to an acutely hospitalized cardiovascular population and demonstrates not only measurement validity but also significant prognostic relevance for short-term mortality [10,13,14]. This distinction is clinically important, as hospitalized patients represent a higher-risk and more functionally vulnerable population in whom early frailty detection may have immediate therapeutic implications.

### 4.3. Prognostic Implications

We observed that gait speed and sit-to-stand performance were strongly associated with frailty status and contributed to a model with good apparent discriminative ability (AUC = 0.83), although the relatively small sample size warrants external validation. Because the frail subgroup was relatively small, the confidence interval around the AUC was wide; therefore, external validation in larger cohorts is warranted. These findings are consistent with previous research showing that gait speed is strongly associated with adverse outcomes, including mortality, in older adults and cardiovascular patients

Because frailty was defined using SPPB scores, the inclusion of its individual components (gait speed and sit-to-stand time) in the regression model may introduce circular reasoning. Therefore, this analysis should be interpreted as exploratory and descriptive rather than as an independent analytical model.

The observed association between frailty and increased 30-day mortality further underscores the importance of functional status as a prognostic marker. Importantly, frailty defined using automated eSPPB was also associated with increased short-term mortality, supporting the prognostic validity of the automated assessment. Although the magnitude of the association was slightly lower than that observed with manual SPPB, the direction and clinical relevance of the findings were consistent. These results suggest that the prognostic value of the SPPB is preserved when assessed using an automated digital system, although confirmation in larger cohorts is warranted.

Frailty likely reflects diminished physiological reserve and reduced resilience to acute illness, increasing vulnerability to adverse outcomes during and after hospitalization. Given the small number of mortality events, estimates are imprecise and should be interpreted with caution. The observed association should be considered exploratory and hypothesis-generating rather than definitive, and requires confirmation in larger multicenter cohorts.

We did not directly compare the prognostic performance of manual and automated SPPB due to the limited number of events. Future studies with larger cohorts should evaluate whether eSPPB provides equivalent or superior prognostic discrimination compared with the conventional manual assessment. Additional analyses comparing AUCs could not be robustly performed due to the low number of events.

Automated frailty assessment using eSPPB may provide a practical and scalable approach for identifying high-risk patients early during hospitalization. Early identification of frailty could support clinical decision-making by informing risk stratification, discharge planning, and targeted interventions such as early mobilization or rehabilitation.

Furthermore, automated assessment may facilitate standardized frailty screening without requiring specialized training or additional staff resources. This may improve feasibility of routine frailty assessment in real-world clinical settings.

However, further studies are needed to evaluate whether implementation of automated frailty assessment leads to improved clinical outcomes.

### 4.4. Limitations

Several limitations should be considered. First, this study was conducted at a single tertiary care center, which may limit generalizability to other healthcare settings. Second, the study population was heterogeneous, including patients with a range of cardiovascular conditions. Differences in baseline risk and disease severity across these groups may have influenced both frailty status and mortality outcomes.

Third, the sample size was modest, and the limited number of mortality events may increase the risk of effect size overestimation and model instability; therefore, these findings should be interpreted as exploratory.

Due to the limited number of events, multivariable adjustment for potential confounders was not feasible. Therefore, the observed association between frailty and mortality may be influenced by residual confounding, including age, comorbidities, and underlying cardiovascular conditions.

Additionally, the SPPB primarily evaluates physical performance and does not capture other important dimensions of frailty, such as cognitive, nutritional, or psychosocial factors. Future studies should explore integration of automated physical performance measures with multidimensional frailty assessments. Larger multicenter studies are required to confirm the magnitude of the observed association.

## 5. Conclusions

In conclusion, automated eSPPB assessment demonstrated good agreement with manual SPPB and was significantly associated with short-term mortality in hospitalized cardiology patients. These findings suggest the validity and potential clinical utility of automated frailty assessment as a scalable tool for functional evaluation and risk stratification in acute cardiology settings. However, given the limited number of events and the single-center design, these results should be interpreted with caution and confirmed in larger multicenter studies before routine clinical implementation.

## Figures and Tables

**Table 1:** Baseline characteristics and physical performance according to frailty status.

Frailty Group	n (%)	Age (Mean ± SD)	% Female	Gait Speed (m/s)	Sit-to-Stand (s)
Frail	17 (15.0%)	79.1 ± 10.5	75.0%	0.52 ± 0.10	22.8 ± 6.0
Pre-frail	60 (53.1%)	76.7 ± 11.2	46.7%	0.85 ± 0.18	16.7 ± 4.5
Non-frail	36 (31.9%)	68.4 ± 12.1	46.7%	1.03 ± 0.21	13.2 ± 3.2

**Table 2:** Clinical and functional variables associated with frailty status.

Variable	β Coefficient	*p* -Value	Interpretation
Age	+0.08	0.21	Older age is associated with higher likelihood of frailty
Gait Speed (m/s)	–2.5	<0.001	Lower gait speed is associated with higher likelihood of frailty
Sit-to-Stand Time (s)	+0.14	0.06	Longer sit-to-stand time is associated with higher likelihood of frailty
Gender (Female = 1)	+0.45	0.12	Not significantly associated with frailty status, although a mild trend was observed

**Table 3:** Association between frailty and 30-day mortality.

Group	Total Patients	Deaths (30-Day)	Mortality Rate (%)
Frail (SPPB < 5)	17	3	17.6% *
Not Frail (SPPB ≥ 5)	96	2	2.1%

* *p* = 0.009 a statistically significant difference in mortality based on frailty status.

## Data Availability

The original contributions presented in this study are included in the article. Further inquiries can be directed to the corresponding author.
